# Diverse Repertoire and Relationship of Exopolysaccharide Genes in Cold-Adapted *Acinetobacter* sp. CUI-P1 Revealed by Comparative Genome Analysis

**DOI:** 10.3390/microorganisms11040885

**Published:** 2023-03-29

**Authors:** Ifra Ferheen, Zaheer Ahmed, Wadi B. Alonazi, Alex Pessina, Muhammad Ibrahim, Sandra Pucciarelli, Habib Bokhari

**Affiliations:** 1 Department of Biosciences, COMSATS University Islamabad, Islamabad 44000, Pakistan; 2 Department of Nutritional Sciences and Environmental Design, Allama Iqbal Open University, Islamabad 44000, Pakistan; 3 Health Administration Department, College of Business Administration, King Saud University, Riyadh 11587, Saudi Arabia; 4 Department of Biotechnology and Biosciences, University of Milano-Bicocca, 20126 Milan, Italy; 5 Department of Biosciences, COMSATS University Islamabad, Sahiwal Campus, Sahiwal 55000, Pakistan; 6 School of Biosciences and Veterinary Medicine, University of Camerino, 62032 Camerino, Italy; 7 Department of Microbiology, Kohsar University Murree, Murree 47150, Pakistan

**Keywords:** cold-adapted microbes, *Actinobacteria* spp., genome-wide analysis, exopolysaccharide, cryoprotectant activity

## Abstract

This study focused on the exploration of microbial communities inhabiting extreme cold environments, such as the Passu and Pisan glaciers of Pakistan, and their potential utilization in industrial applications. Among the 25 initially screened strains, five were found to be suitable candidates for exopolysaccharide (EPS) production, with strain CUI-P1 displaying the highest yield of 7230.5 mg/L compared to the other four strains. The purified EPS from CUI-P1 was tested for its ability to protect probiotic bacteria and *E. coli* expressing green fluorescence protein (HriGFP) against extreme cold temperatures, and it exhibited excellent cryoprotectant and emulsification activity, highlighting its potential use in the biotechnological industry. Furthermore, the genome of *Acinetobacter* sp., CUI-P1 comprised 199 contigs, with a genome size of 10,493,143bp and a G + C content of 42%, and showed 98.197% nucleotide identity to the type genome of *Acinetobacter baumannii* ATCC 17978. These findings offer promising avenues for the application of EPS as a cryoprotectant, an essential tool in modern biotechnology.

## 1. Introduction

Microbes are among the most significant and abundant living things in our ecosystem, making them the gold mine of “Discovery” due to their extreme genetic and metabolic diversity [1]. Himalayan mountains are characterized by shallow temperature, which is a suitable environment for growing cold-adapted microbes and can be explored for their various beneficial usages [2]. Microbes residing in frozen habitats are subjected to numerous freeze and thaw cycles and hence are well adapted to such transitions due to their unique physiology and surface properties [3]. Numerous bacteria have been isolated from the cold environment, including *Pseudomonas*, *Exiguobacterium*, *Planococcus*, *Staphylococcus*, *Janthinobacterium*, *Arthrobacter*, *Pseudoalteromonas*, *Actinobacteria*, *Marinobacter*, and *Paenibacillus* [3,4,5,6]. Acinetobacter species are Gram-negative, coccoid rods that can be challenging to detain; these bacteria are found in various environments, including soils, freshwater, oceans, sediments, and contaminated sites, making them ubiquitous in nature. Their adaptable metabolic characteristics allow them to break down a broad range of natural compounds, actively participating in the nutrient cycle within the ecosystem. Given the ecological and clinical significance of the genus, Acinetobacter is a valuable microorganism for environmental microbiological investigations, pathogenicity assessments, and industrial chemical production [7]. Additionally, Acinetobacter species have been reported to possess genes related to multi-drug-resistance mechanisms, including those involved in EPS production and β-lactamase acquisition, as well as transposon insertions, mutations in porins, and changes in efflux pumps [1,8]. The varying potential to survive such an extremely cold environment has been attributed to the production of various multitalented macromolecules such as proteins and exopolysaccharides, which provide protective sheathing to bacteria to cope with extreme ecological niches [9,10,11]. Exopolysaccharide (EPS) carbohydrate polymers produced by many bacteria to form an external capsule are also known for their cryoprotectant activity by forming an organic network within the ice, along with modifications in the structure of brine channels, contributing to the enrichment and retention of psychrophilic microbes in ice crystals [12]. EPSs are macromolecules with high molecular weight and are carbohydrates in nature, and offer specific attributes and functions beneficial to microbes [6]. This property of extremophile bacteria owing to exopolysaccharides (EPSs) has also opened opportunities in various industrial applications; EPSs extracted from bacterial sources possess great diversity in terms of properties and are significantly different from EPSs originating from plants [13]. Microbial polysaccharides, particularly EPS, represent an untapped market with the potential to be produced by a variety of microorganisms including bacteria, molds, and yeasts [14]. EPS are regarded as crucial and extensively researched types of microbial polysaccharides, exhibiting significant potential as bioactive molecules with proven immunostimulatory, anti-tumor, antioxidant, antiulcer, and cholesterol-reducing properties [15,16].

Moreover, EPSs also provide a promising role in biotechnology; these can enhance the thermostability of proteases and many other biotechnologically necessary enzymes [6,17]. The exopolysaccharides produced from various sources have been reported to possess excellent physicochemical properties, so they are used in various industries, including food, pharmaceutical, medicine, etc. [18]. EPS is an ideal natural ligand source as it provides binding sites for charged particles, including heavy metals; that is why it is widely employed in the bioremediation of heavy metals, i.e., lead, cadmium, nickel, etc. [19].

Several prior studies have shown the potential ability of extracellular polymeric substances (EPS) to protect against freezing in cold and icy environments. For instance, EPS from a *Pseudomonas* sp. found in Antarctica was observed to provide cryoprotection to the producing strain [4]. Moreover, the cryoprotective role of EPS has also been documented in cyanobacteria and microalgae inhabiting extremely cold environments [20]. These studies collectively support the notion that EPS is an important factor that enables bacterial survival in harsh environments. However, that cryoprotectant role is for that bacteria or wither in caging of the cyanobacteria and microalgae. To the best of our knowledge, the cryoprotective properties of exopolysaccharides (EPS) derived from extreme cold habitats have not been documented for bacterial strains that encapsulate probiotics. Furthermore, the use of such EPS has been shown to enhance the shelf life of HriGFP protein confined within *Escherichia coli* strains.

Considering the aforementioned research gaps, we conducted an investigation to characterize the novel exopolysaccharide (EPS)-producing bacteria from the previously unexplored niches of Passu and Pisan Glaciers, namely *Acinetobacter* sp. The study aimed to determine the physical and functional attributes of this bacterial strain and evaluate its industrial potential. In addition, we performed draft genome sequencing to gain a better understanding of the factors contributing to EPS production and genetic diversity. Relevant functional genes were also analyzed to identify potential industrial and environmental applications of this novel strain.

## 2. Materials and Methods

### 2.1. Sample Collection 

Glacier samples were collected in sterilized bottles from the Passu (36°27′26″ N 74°51′48″ E) and Pisan (36°12′52″ N 74°30′61″ E) glaciers of Pakistan; in November 2017. Samples from different points in triplicate were collected and stored in RNA later for subsequent nucleic acid studies and were transported to the laboratory under sterile and dark conditions at 4 °C, and further analyses were performed within 24 h. Samples collected from each point were filtered by using a cellulose nitrate filter with a 0.45 μm diameter pore size (Sartorius Stadium Biotech, Göttingen, Germany), and the filters were incubated for pre-enrichment into Luria Bertani (LB) agar plates and incubated at 4 °C for 72 h to isolate bacteria. Single colonies with significantly different morphology in terms of their color, margins, shape (convex and concave), and sizes were grown further for purification.

### 2.2. Screening and Isolation of EPS-Producing Bacteria 

Twenty-five isolates were inoculated and screened for EPS production using LB agar medium enriched with 5% of saccharides, including sucrose, glucose, arabinose, lactose, and maltose. All media were autoclaved at 121 °C for 15 min, and sugars were autoclaved separately. A total of 10 µL of the fresh overnight culture of each pure strain was inoculated on the saccharides-enriched LB medium and spread on a plate using a sterile cotton bud. After three days of incubation, plates were observed for rugose, mucoid, and ropy colonies to check EPS production.

EPS-producing psychrophilic microbes were initially observed under a BX41 System Microscope for visualizing body shape, size, behavior, and other morphological features. API 20E (BioMerieux, Marcy-l’Étoile, France) and API 20NE were used for the analytical profile index of EPS-producing psychrophilic bacteria. Out of 25 isolates, only five strains (1A, 1B, 2D, 4C, and CUI-P1) produced EPS and were further identified by 16S rRNA gene sequencing. For species identification, genomic DNA was extracted from isolates by using a WizPrep DNA extraction kit and subjected to PCR using 16S rRNA gene universal primers Ulf (5′-CGGTTACCTTGTTACGACTT-3′) and U3R (5′-CAGCAGCCGCGGTAATAC-3′) by using following conditions: 3 min for denaturation at 95 °C, 30 cycles of 30 s at 95 °C, 30 s for annealing at 55 °C, 60 s for elongation at 72 °C, and a final extension at 72 °C for 10 min (Thermocycler, Bio-Rad, Hercules, CA, USA). PCR products were run on 1% agarose gel at 100 V for 30 min and a 1 kbps ladder (Thermofisher Scientific, Waltham, MA, USA). PCR products were purified by using PCR and Gel band Purification kit (GRISP, Porto, Portugal) and sent to Eurofins Genomics (Ebersberg, Germany) for Sanger sequencing. All sequence alignments were conducted by a blast search in the NCBI database and phylogenetic analysis was performed by using MEGA11 software (Molecular Evolutionary Genetic Analysis). A Kimura two-parameter model (K2P) was used to determine the model of nucleotide substitution that best fit the data.

### 2.3. Extraction and Purification of EPS

Extraction and purification of bacterial polysaccharides were performed using the previously described method [21]. For EPS production from strains, LB broth enriched with 5% sucrose was prepared, and inoculums at a rate of 0.5% were inoculated in baffled flasks to ensure aerobic conditions and were incubated at 4 °C, shaking at 150 rpm for five days. After incubation, the broth was heated at 100 °C for 10 min to dissolve cell-attached molecules of EPS, followed by centrifugation at 12,000 rpm at 4 °C for 20 min to get rid of particulate material and cell debris to obtain clear supernatant. Ethanol was chilled at −20 °C, and 1:1 volume was added to precipitate crude exopolysaccharide present in the supernatant. It was again centrifuged under the conditions mentioned above, and the EPS in the form of a pellet was obtained. After dissolving in a small amount of water, dialysis of crude exopolysaccharide was performed using 12,000–14,000 Da dialysis tubing against double distilled water for 24 h.

The resultant solution was precipitated by adding the chilled ethanol, and after that a pellet was obtained by centrifugation at 4 °C and 12,000 rpm for 20 min and lyophilized. The freeze-dried pellet was weighed, and the yield of EPS was measured. To further purify EPS from any coexisting protein debris, EPS was dissolved in a small amount of H_2_O with pH adjusted to 4 by using 14% trichloroacetic acid (TCA) and incubated for 10–12 h at 4 °C, followed by centrifugation at 4 °C and 12,000 rpm for 20 min. The pellet was discarded, and the supernatant was adjusted to neutral pH. Exopolysaccharide was again precipitated by adding an equal volume of chilled ethanol, and the obtained pellet was lyophilized using the freeze drier. The protein content in the EPS sample was measured by binding Coomassie Brilliant Blue G-250 to protein, using bovine serum albumin as a standard [22].

### 2.4. Cryoprotectant Activity of EPSs

A probiotic *Lactobacillus* sp., previously isolated in our lab, was used to determine the comparative cryoprotective activity of all five extremophile-derived EPSs, including CUI-P1 *Acinetobacter* sp. All the strains were grown on a nutrient agar plate initially, and a single colony of each strain was picked and inoculated separately in 10 mL of nutrient broth medium. The purified exopolysaccharides produced by all five strains: CUI-P1 (*Acinetobacter* sp.)*,* 1A (*Exiguobacterium indicum*), 1B (*Hymenobacter* sp.), 2D (*Staphylococcus lantus*), and 4C (*Sphingomonas* sp.) were tested for their comparative cryoprotective role. Three Eppendorfs were selected, i.e., the 1st as a negative control (exclusively 500 µL of *Lactobacillus* sp.), the 2nd as a positive control (500 µL *Lactobacillus* sp. + 500 µL of 10% glycerol), and the 3rd as a test (500 µL of *Lactobacillus* sp. + 500 µL of 10% exopolysaccharide). All Eppendorfs were incubated at −20 °C and proceeded with 20 repeated freeze and thaw cycles over 72 h afterward; cultures were thawed and serially diluted up to the 8th dilution. A total of 10 µL of the 8th dilution of each Eppendorf was spread on a nutrient agar plate and incubated at 37 °C for 24 h, and CFUs were calculated. Moreover, as a rapid assay, the biological activity or fluorescence emission of green fluorescent protein HriGFP [23] expressed in *E. coli* was evaluated after caging it with the EPS cryoprotectant of all five strains. In an Eppendorf, 200 µL of HriGFP-induced cells were mixed with EPS isolated from psychrophilic bacteria and incubated at −20 °C and proceeded with 20 freeze and thaw cycles for temperature fluctuations in 72 h. Eppendorfs were visualized by using a UV trans-illuminator to determine the caging cryoprotectant activity of EPS. The EPS with the best cryoprotective role was studied in detail for its physicochemical properties. Since *Acinetobacter* sp. polysaccharide, i.e., EPS from CUI-P1 depicted the best cryoprotectant role; therefore, CUI-P1 EPS was characterized using the following methods.

### 2.5. Emulsification Stability

To check the emulsification stability of shortlisted *Acinetobacter* sp. 0.5 mg of EPS was added to 0.5 mL of deionized water by heating at 50 °C for 10 min, and the volume was raised to 2 mL by adding sterile PBS and homogenizing it by vortexing. This step was followed up by adding 0.5 mL of hexadecane, and optical density at 540 nm was measured initially (Ao) and then at an interval of 30 min (At). Emulsification activity was calculated in percentage as described previously [21].

Emulsification activity= At/Ao × 100

### 2.6. Metal Tolerance of EPS-Producing Bacteria

Three heavy metals, mercury, arsenic, and iron, were tested by using the plate-diffusion method to assess the tolerance enhancement of three heavy metals by exopolysaccharide (EPS) production. LB agar medium was utilized, and metal concentrations ranging from 500 to 10,000 ppm were incorporated, along with 5% sucrose enrichment. A control medium devoid of sucrose was also prepared. Subsequently, 20 µL of an overnight bacterial culture was inoculated on both the experimental and control media, followed by incubation at 4 °C for 48 h. Bacterial growth on sucrose-positive and sucrose-negative was monitored and compared as an indicator of metal tolerance enhancement.

### 2.7. Physical Characterization of EPS

Scanning electron microscopy was used for the structural characterization of exopolysaccharides. Isolated EPS from *Acinetobacter* sp. was fixed on an aluminum stub and gold-sputtered and examined through SEM by maintaining an accelerated voltage of 10 kV.

Purified EPS from *Acinetobacter* sp. was dissolved entirely in double distilled autoclaved water (1 mg/mL) with continuous stirring for 60 min under a nitrogen stream. When the sample attained room temperature, EPS was diluted up to 0.01 mg/mL, and 5 mL of it was homogeneously distributed on a mica sheet which acts as a carrier medium. Atomic force microscopy images were taken by scanning probe microscope in tapping mode, and cantilever oscillation was set at a frequency of 158 kHz with a driven amplitude of 0.430 V.

The X-ray diffraction pattern of EPS of CUI-P1 was recorded using Bruker D8 Quest X-ray powder diffractometer (Bruker AXS, Madison, WI, USA) equipped with Bragg Brentano HD optics, a sealed tube copper X-ray source (λ = 1.54178 Å) and a PixCel3D Medipix detector. The configuration included a power of 40 kV × 40 mA, a 1.0 mm divergence slit, a 1.0 mm anti-scattering slit, a 0.1 mm detector slit, and a 0.6 mm receiving slit. Data were collected between 10° and 90° in 2θ using the Analytical Data Collector software (Malvern Panalytical’s XRD software, X’Pert HighScore Plus).

### 2.8. Chemical Characterization of EPS 

The primary structural groups of refined exopolysaccharides were detected using Fourier-transformed infrared spectroscopy [13]. Briefly, the lyophilized exopolysaccharides were mixed in KBr (Potassium Bromide) at a ratio of 1:100 and later converted to fine pellets by applying pressure. Spectra were recorded on FTIR in a region of 4000–400 cm^−1^ in 16 scans.

The monomer composition of the EPS isolated from *Acinetobacter* sp. Was further characterized by using the high-performance liquid chromatography method as described previously [24]. Briefly, Isolated EPS was hydrolyzed in 2M trifluoroacetic acid (TFA) and the solution was incubated in Eppendorf ThermoMixer^®^ at 95 °C for 3 h. Afterward, samples were evaporated to dryness using a Concentrator plus/Vacufuge^®^ plus at 45 °C. Finally, samples were dissolved in 0.250 to 1 mL of ultra-pure water and the solutions were vortexed for 10 s. Monosaccharides analysis was performed in reversed-phase C18 columns on an HPLC system equipped with a UV-VIS detector, adjusted on wavelength 245 nm. The mobile phase consisted of a gradient program along with solvent A (10% acetonitrile) in 0.1 mol L^−1^ ammonium acetate (Fluka) buffer pH (5.5) and solvent B (25% acetonitrile in 0.1 mol L^−1^ ammonium acetate buffer pH 5.5). The first 55 min gradient of 30 to 100% buffer B and the next 55–65 min 100–30% gradient was used for separation. A total of 5 min of initial conditions was applied to re-equilibrate the column for further analysis. A total of 20 μL of EPS sample was injected into the chromatograph, and results were compared with saccharides standards, i.e., D-glucose (Glc), D-galactose (Gal), L-rhamnose (Rha), D-mannose (Man), D-arabinose (Ara).

### 2.9. Genomic DNA Extraction, Genome Sequence, and Analysis

A pure culture of CUI-P1 was grown in LB broth at 4 °C for 72 h, and the genomic DNA was extracted from the culture using the Genomic DNA Isolation Kit (WizPrep DNA extraction kit). The concentration and purity of the extracted DNA were determined using a NanoDrop 2000 spectrophotometer (Thermo Fisher Scientific, Waltham, MA, USA). The DNA was sequenced using Illumina MiSeq sequencing. According to the manufacturer’s protocol, the library was prepared using a TruSeq Nano DNA kit (Illumina, Inc., San Diego, CA, USA), and sequencing was performed in a MiSeq 2 × 250 bp run. De novo assembly of the processed reads was performed using Geneious Prime (3) with default settings. All four genomes were annotated using the RAST annotation system using SEED viewer to predict rRNAs, tRNA, coding genes, and GC contents compositions. The Kyoto Encyclopedia of Genes and Genomes (KEGG) and Clusters of Orthologous Groups of proteins (COGs) were used for the classification of the predicted genes, i.e., virulent genes, various secretion systems, drug-resistant genes, and different pathways [25]. SignalP server and TMHMM server were used to predict genes’ signal peptides and transmembrane helices [26,27]. The genome alignment of Ecc strains was conducted using the MAUVE software package [26], which is employed to conduct an alignment of multiple genomes to look at the highly similar subsequences, and evolutionary events such as inversions rearrangement and to reveal the correct global alignment. The seven genomes were also compared using the CGView tool [28]. A circular genomic map of Ecc strains was generated using CGView that represents circular genomes into a graphical map resulting in base composition plots, sequence features, analysis of GC skew, GC contents, and RNA number.

Comparison of *Acinetobacter* sp. strain CUI-P1 with reference strain *Acinetobacter baumannii* ATCC 17978 was conducted using CGView. Conserved regions were trimmed by using trimAl. Putative virulence-associated genes in the draft genomes of the *Acinetobacter* sp. strain CUI-P1 strains resourced from various niches were analyzed using reference strains at RAST and SEED viewer [29,30]. The presence or absence of EPS and virulent and drug-resistant coding sequences were retrieved using *Acinetobacter baumannii* ATCC 17978 genes as bait sequences following independent confirmation by performing nucleotide BLAST analysis at NCBI as BioEdit. The genes with query coverage higher than 70% and similarities higher than 50% were taken as homologs.

### 2.10. Statistical Analysis

All assays and experiments conducted in this study were performed in triplicates, and the results were expressed as mean and standard deviation. The student’s *t*-test evaluated a significant difference, and the <0.05 *p*-value was considered statistically significant. To perform inferential statistics, a one-way ANOVA test was conducted to determine if there is a significant difference in means of EPS production and cryoprotectant activity of different bacterial strains followed by a post hoc analysis, performed by using Tukey’s HSD test to determine significant strains by using the ORIGIN-PRO 2019 software.

## 3. Results

### 3.1. Screening of Isolated Strains for EPS Production 

**Figure 1:**
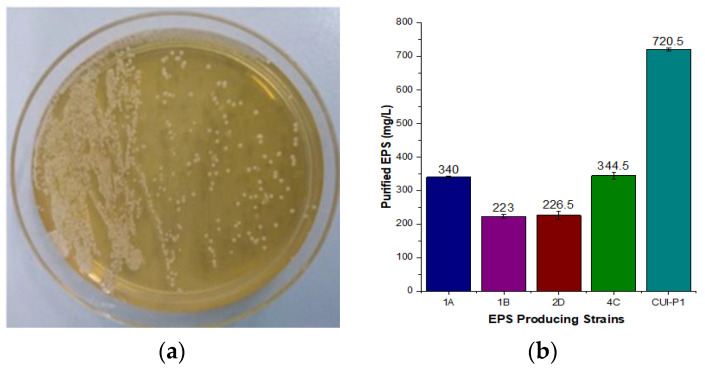
(**a**) Colony morphology of EPS producing CUI-P1 *Acinetobacter* sp. and (**b**) EPS yield of five cryoprotectant strains isolated from Passu and Pisan glaciers of Pakistan.

We investigated bacterial isolates collected from the Passu and Pisan Glaciers of Pakistan, sites not explored before for any such study. In the present study, all psychrophilic microbes were screened for EPS production by growing them on an LB medium enriched with 5% sucrose and incubated at 20 °C; mucoid colonies were observed, reflecting EPS production by all strains. The isolates that gave mucoid and ropey behavior were considered EPS-producing (Figure 1a). Out of 25 isolates, only five strains (1A, 1B, 2D, 4C, and CUI-P1) produced EPS. Among five EPS-positive strains, EPS production by strain CUI-P1 was approximately 720.5 mg/L, whereas the other four produced EPS from 220 to 340 mg/L under the same conditions (Figure 1b). One-way ANOVA test on the data to determine if there is a significant difference in the mean EPS production among the different strains (1A, 1B, 2D, 4C, and CUI-P1). The results show that there is a statistically significant difference in the mean EPS production among the strains (F(4, 15) = 285.82, *p* < 0.0001). Post hoc analyses with Tukey’s Honestly Significant Difference (HSD) test revealed that the EPS production of the CUI-P1 strain was significantly higher than all the other strains (*p* < 0.0001). Therefore, results suggested significant differences in EPS production among the different strains, with CUI-P1 and 4C strains having the highest EPS production.

### 3.2. Identification of EPS-Producing Strains

Out of 25 total isolates, five depicted exopolysaccharide production (Table 1). These five isolates were identified using biochemical and molecular methods. After 16S rRNA sequencing, these strains were identified as 1A *Exiguobacterium indicum*, 1B *Hymenobacter* sp., 4C *Sphingomonas* sp., 2D *Staphylococcus lantus*, and CUI-P1 *Acinetobacter* sp., and submitted to NCBI GenBank under the codes MN556305, MN556616, MN556615, MN556340, and MN556614, respectively.

### 3.3. Cryoprotectant Role of Isolated EPS

**Figure 2:**
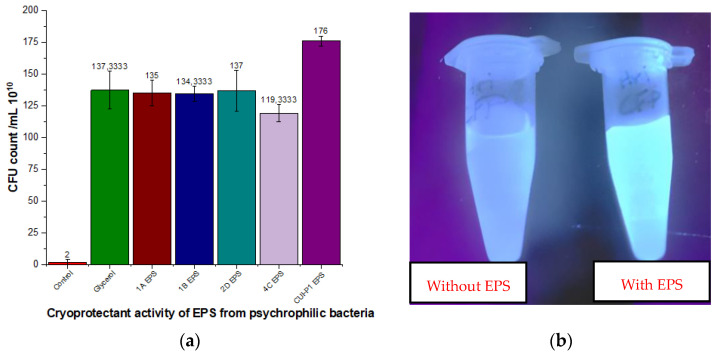
(**a**) Cryoprotectant activity of EPS isolated from psychrophilic bacteria in comparison with glycerol and control. (**b**) Cryoprotective role for CUI-P1 EPS in increasing the shelf life of *E. coli* strain caging HriGFP protein.

Five strains were further screened for their cryoprotective capability. The findings of cryoprotective activity revealed that exopolysaccharides from all the strains had the potential to act as a shield to protect bacteria in an adverse cold environment. The standard plate count of negative control depicted that not even a single colony of *Lactobacilli* was able to survive. The positive control and the intervention group showed more or less similar results. All five tested exopolysaccharides are depicted in Figure 2a. The control and intervention groups underwent twenty freeze and thaw cycles. EPS isolated from *Acinetobacter* sp. exhibited a maximum CFU count of 180 × 10^10^. In positive control (20% Glycerol as a protectant) CFU count of 150 × 10^10^ was observed. Exopolysaccharide produced by *Acinetobacter* sp. exhibits better activity than the positive control (20% Glycerol) and the EPS produced by the other four strains. For the one-way ANOVA, the F value is significant at *p* < 0.05 (F = 25.47, df = 6, 14, *p* < 0.001), indicating that there is a significant difference in cryoprotectant activity between the different treatments. Moreover, Tukey’s HSD analysis results confirm that any q-value that is greater than the critical value indicates that the difference between the means of the two groups is significant at the 95% confidence level. The mean CFU count for the control group was significantly different from the mean CFU count for all other groups: glycerol, 1A EPS, 1B EPS, 2dD EPS, 4C EPS, and CUI-P1 EPS. The q-values for each of these comparisons were all greater than the critical value of 3.05.

As far as the cryoprotective response of HriGFP expressing *E. coli* is concerned, it was revealed that all the five different kinds of EPS produced have shown preservative impact by enhancing their shelf life, but exopolysaccharide from *Acinetobacter* sp. was best among all by increasing shelf life up to three days. Compared to negative control after 48 h incubation at −25 °C followed by temperature fluctuations, fluorescence was maintained in Eppendorfs containing EPS. However, amazingly, EPS isolated from *Acinetobacter* sp.-maintained fluorescence HriGFP for 72 h and depicted higher caging ability than EPS isolated from other four sources, i.e., 1A, 1B, 2D, and 4C. EPS (Figure 2b). *Acinetobacter* sp. produced maximum EPS, which also exhibited a superior response when tested for its cryoprotective role for strain survival and protein activity assay HriGFP. EPS from *Acinetobacter* sp. structure was physiochemically characterized in detail. Moreover, it was also tested for other functional properties, including emulsification and metal tolerance roles.

### 3.4. Emulsification Stability

Emulsification activity was determined by incubating 0.5 mg of EPS isolated from *Acinetobacter* sp. in a reaction mixture containing deionized water, PBS, and Hexadecane. The results showed that purified fractions EPS from *Acinetobacter* sp. retained 98%, 91%, 86%, and 79% of the emulsification activity after 30, 60, 90, and 20 min time intervals, respectively (Table 2). Results depicted that EPS isolates from psychrophilic *Acinetobacter* sp. have strength in retaining the emulsion of hydrocarbon in water compared to the standard solution (without EPS) emulsion breaks rapidly within an initial incubation of 30–60 min. The data were analyzed by a one-way ANOVA test followed by a post hoc Tukey HSD test, which showed a significant difference between the emulsifying activity of the EPS and the standard emulsifier (*p* < 0.05). These findings suggest that the EPS has a superior emulsifying activity compared to the standard emulsifier.

### 3.5. Metal Tolerance

**Figure 3:**
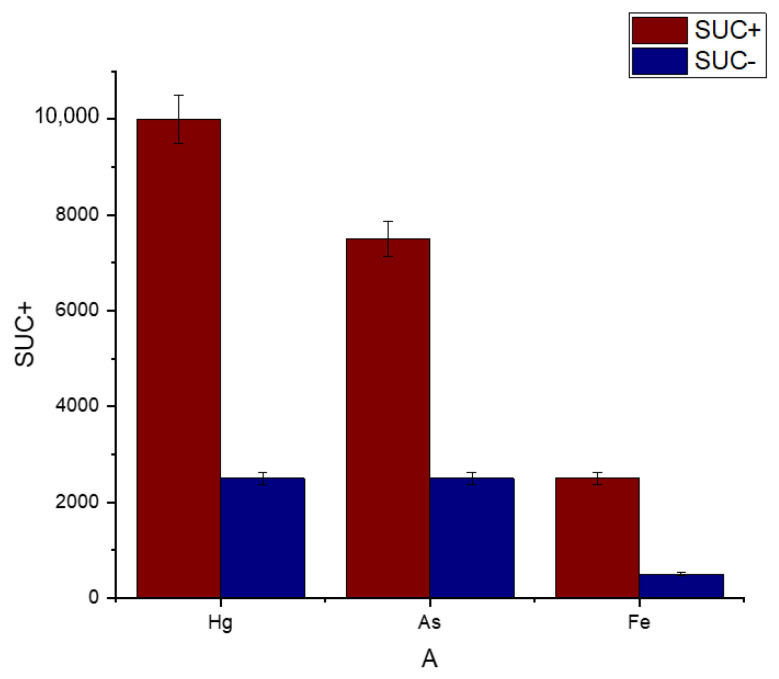
High metal resistance of EPS producing CUI-P1 *Acinetobacter* sp.

To check the metal tolerance activity of *Acinetobacter* sp., it was grown on the LB agar plate with different heavy metal concentrations (enriched and non-enriched 5% sucrose). The result depicted that *Acinetobacter* sp. survived much better on LB agar containing 5% sucrose and a high heavy metal concentration than on control media (without sucrose). In the media where sucrose was present, bacteria were able to produce exopolysaccharides and talented to tolerate mercury, arsenic, and iron up to 10,000, 7500, and 2500 ppm, respectively, which was significantly higher than metal tolerance in LB medium without sucrose (Mercury 2500, Arsenic 2500, and Iron 500 ppm). Interestingly, it was found that when psychrophilic bacteria have an EPS shield around them, they tolerate mercury up to 10,000 ppm, arsenic at 7500 ppm, and iron at 2500 ppm, which is significantly high resistance when compared with bacteria without a shield; resistance was in the order Hg > As > Fe (Figure 3). To analyze the statistical significance of metal tolerance between SUC+ and SUC− strains for Hg, As, and Fe, a two-sample t-test can be performed. The *p*-values for all three metals were less than 0.05, indicating that there is a significant difference in metal tolerance between the SUC+ and SUC− strains. Specifically, the strain in presence of sucrose has a higher metal tolerance than the SUC− strain. These results suggest that the EPS produced by strain CUI-P1 may play a role in metal tolerance.

### 3.6. Chemical Characterization of EPS

**Figure 4:**
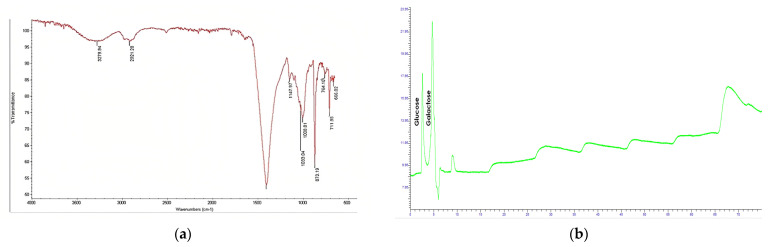
(**a**) Fourier transform infrared (FTIR) spectrum of EPS isolated from *Acinetobacter* sp. (**b**) Monomer composition analysis of CUI-P1 exopolysaccharides by HPLC.

The FTIR spectra of the EPS-P1 have been depicted in Figure 4a. A variety of peaks were observed, ranging from 3279 to 666 cm^−1^. The vast stretch at 3279.94 cm^−1^ has been attributed hydroxyl group from polysaccharides [31], and the peak at 2925 cm^−1^ has been attributed to C-H [32]. Peaks between 1000 and 1200 cm^−1^ are confirmatory peaks of the polysaccharide nature of the material [33,34]. Stretching at 1008.81 cm^−1^ indicates C-O linkage from the alcohol group, whereas the small peak at 1147.97 has been attributed to asymmetric stretching owing to the O-C-O group [13]. The characteristic extended at 1454.12 cm^−1^ was allied to the symmetrical COO-link vibrations [35].

HPLC chromatogram results were compared with monosaccharides standards peaks, i.e., D-glucose (Glc), D-galactose (Gal), L-rhamnose (Rha), D-mannose (Man), and D-arabinose (Ara) (Figure 4b). HPLC analysis revealed that EPS from *Acinetobacter* sp. was composed of glucose and galactose. The peaks depicted retention patterns that matched glucose and galactose depicting its heteropolysaccharide nature.

### 3.7. Physical Characterization of EPS 

**Figure 5:**
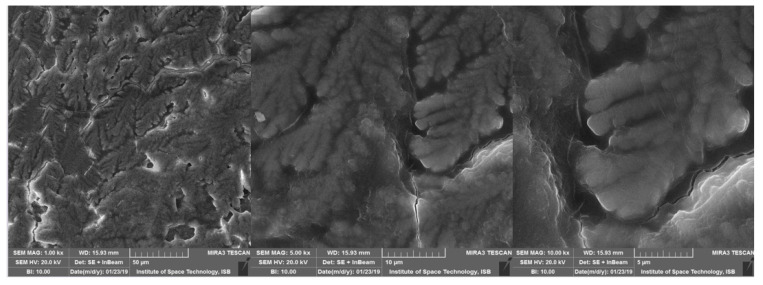
Scanning electron microscopy images of CUI-P1 EPS molecules at 1.00 k×, 5.00 k× and 10.0 k×.

SEM was used to examine the exterior morphological feature of isolated EPS from *Acinetobacter* sp. (Figure 5). At 500 and 1000×, exopolysaccharides gave an impression of a rough and uneven surface. At higher resolutions, i.e., at 5000 and 10,000×, it seems shinier and more compact, and the structure seems like the kernel of a walnut. White patches and lines can also be seen on the surface of EPS-P1. Overall, the structure of EPS seems compact when seen on higher resolution.

**Figure 6:**
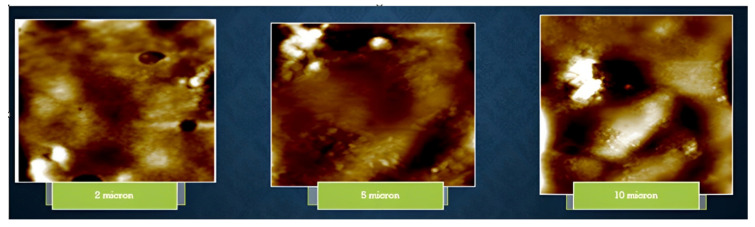
AFM images of CUI-P1 EPS molecules.

AFM results of isolated EPS were obtained by dissolving 0.01 mg of EPS in 1 mL of deionized water, and 5 mL of it was homogeneously distributed on a mica sheet. Results depicted spherical and rounded lumps, which are tightly packed, portraying pseudoplastic properties of EPS as it illustrates a solid affinity of water (Figure 6).

**Figure 7:**
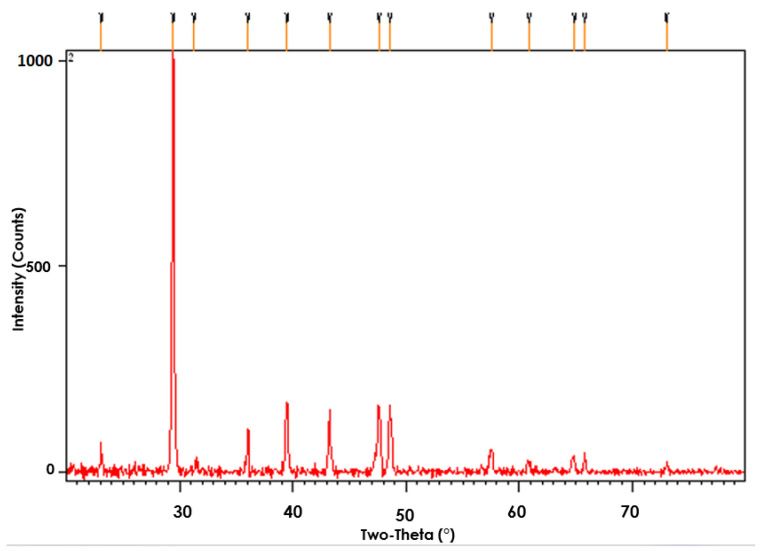
XRD Diffractogram of CUI-P1 EPS molecules.

X-ray diffraction is applied to infer crystalline features, crystallite size, and crystallinity of various biomaterials such as exopolysaccharides [13]. The XRD diffractogram of EPS from *Acinetobacter* sp. shown in Figure 7 highlights the sharp peak at 29.2 θ. Most exopolysaccharide sample sizes range in this region, revealing its crystalline nature. Several smaller sharp peaks at 36, 39, 43, 47, and 48.5 θ were also observed. Sharp peaks again confirmed the crystalline structure of exopolysaccharides. The majority of exopolysaccharides reported in the literature have amorphous peak behavior (9), and the XRD patterns of EPS-P1 differ from those of biomaterials. Somehow, similar results have been reported for *Bacillus tequilensis* FR9 which exhibited a mixture of sharp and a little broader peak depicting its crystalline and amorphous structure [36].

### 3.8. Genome Sequencing of Acinetobacter sp. CUI-P1 Provides Genetic Evidence for the EPS Production

The draft genome sequence of *Acinetobacter sp.* strain CU I-P1 consists of 10,492,851 bp with a GC content of 42.9% and an *N*_50_ value of 195,936 bp (Table 3). The mean read coverage for the assembly was 158.0 × 2. The annotation and gene prediction was made using Rapid Annotations using Subsystems Technology (RAST) v.2.0 server [29] and the NCBI Prokaryotic Genome Annotation Pipeline (PGAP) v.4.7 for assembled contigs. This resulted in the identification of 9718 genes comprising 16S rRNAs.

The CUI-P1 genome encodes 227 stress response genes in the *Acinetobacter* sp. strain, among which eight cold shock proteins help the bacterium survive under low temperatures. CUI-P1 genome contains many industrial essential genes/proteins such as glycosyltransferase (GTs), exo-polysaccharide, lipases, and protease. Glycosyltransferase is an enzyme involved in the glycosylation of lipids, proteins, and polysaccharides in bacteria [37].

Glycosyltransferase has also been employed at the industrial level to develop a glycol-conjugated vaccine and glyco-randomized drugs using natural or recombinant bacteria. Strain CUI-P1, which encodes more than 30 types of glycosyltransferase enzymes, can be used as a potential player in drug discovery and vaccine development.

Exopolysaccharides (EPS) are produced by many bacteria, including environmental bacteria, pathogens, and food bacteria [38]. These polymers are used in various industries such as food, pharmaceuticals, petroleum, and cosmetics due to their high viscosity stability and gelling ability. CUI-P1 strain encodes two exopolysaccharide biosynthesis genes that can be explored in the future for its industrial application. Bacterial protease, lipases, and analyses are extensively used as a natural and eco-friendly alternative to food, leather, detergent, medicine, and textile chemicals. CUI-P1 strain encodes 27 genes for lipase production, 76 genes for protease production (belonging to different classes of proteases), and four genes for amylase production. These cold adaptive enzymes of CUI-P1 are stable and functional at lower temperatures and would lead to energy conservation in various industrial setups.

Moreover, *Acinetobacter* sp. strain, CUI-P1, also encodes several genes associated with heavy metals such as Zinc, Copper, Arsenic, Fosfomycin, Tetracycline, Beta-lactamase, Cadmium, and Chromium compounds indicating the strain as bioindicator of the pollutant of these glaciers. Overall, the CUI-P1 strain harbors genes of many industrial and medically essential enzymes and provides us with helpful insight into the microbial diversity of Pisan Glacier Pakistan.

## 4. Discussion

The main findings of the current study are five bacterial strains, i.e., 1A *Exiguobacterium indicum*, 1B *Hymenobacter* sp., 4C *Sphingomonas* sp., 2D *Staphylococcus lantus*, and CUI-P1 *Acinetobacter* sp. following genomes sequence of CUI-P1 *Acinetobacter* sp. which showed significant EPS production. Out of 25 isolated strains, these five bacterial strains were obtained from new niches of Passu and Pisan glaciers, which were identified using biochemical and molecular methods. These findings are of great interest as psychrophilic microbes are involved in efficiently resisting cell rupturing from ice crystals under extremely low-temperature stress. The glacier isolates investigated during the current study could tolerate cold stress. Interestingly, several studies suggest that bacteria adapt two mechanisms to tolerate cold stress and ice crystal damage; they upregulate cold assimilation proteins and modify their cell wall by secreting exopolysaccharides around them which act as a shield [39].

Similarly, psychrophiles produce different polysaccharides in stress conditions that help them cope with the extreme environment; thus, EPS provides them resistance against temperature fluctuations, antibiotics, metals, salts, etc. [6]. Moreover, in the current study, EPS isolated from all 5 EPS-positive strains’ cryoprotectant ability was also evaluated. Interestingly, EPS from *Acinetobacter* sp. was able to protect or cage *Lactobacillus* sp. more effectively than EPS isolated from other four sources (EPS-1A, EPS 1B, EPS-4C, EPS-2D). This finding was further examined using fluorescent protein HriGFP expressing *E. coli;* the results revealed that *Acinetobacter* sp. had shown preservative impact by enhancing its shelf life by up to 3 days. Furthermore, previous studies have also reported the cryoprotective potential of EPS isolated from other psychotropic bacteria such as *Colwellia psychrerythraea* 34H & *Janthinobacterium* sp. Ant5 [5,12].

Exopolysaccharide is a biotechnologically important biomaterial produced in response to biotic and abiotic stresses, and is used in the food industry, biomedicine, bioleaching, and cosmetics [17]. Exopolysaccharides from microbes and plants have been known for their emulsifying role; this attribute makes them a potential candidate for the food industry. The emulsification of EPS is determined by retaining the emulsion of hydrocarbons in water [6]. Current emulsification results of EPS from *Acinetobacter* sp. were compared with previous studies in which EPS originated from *Acinetobacter* sp. ID1 retained less than 50% of emulsification activity [4]. Initially, industrial-scale production of exopolysaccharides was dominated by xanthan gum, produced by the bacterial strain *Xanthomonas campestris*, which remains one of the most widely produced polysaccharides in contemporary times [40]. In the literature, different bacterial strains isolated from diversified niches were evaluated for their EPS production; *Pseudoalteromonas* sp. isolated from Madeira archipelago ocean sediments produced 4.4 g/L.

In comparison, EPS isolated from *Lactobacillus kefiranofaciens* showed more or less similar results by retaining 91% and 88% emulsification after 30 and 60 min intervals, respectively. These results depicted that polysaccharides produced by *Acinetobacter* sp. CUI-P1 are potential candidates to be used as an emulsifier.

In the current study, the ability of EPS to enhance metal tolerance from *Acinetobacter* sp. was also explored. Interestingly, EPS from *Acinetobacter* sp. was able to tolerate mercury up to 10,000 ppm, arsenic up to 7500 ppm, and iron up to 2500 ppm, which is significantly a very high resistance compared to bacteria without a shield (strains grown on LB medium supplemented with metals without sucrose). Similar patterns were observed when results were compared with previous studies, i.e., EPS from *Pseudoalteromonas* sp. could tolerate mercury and iron up to 7500 and 500 ppm, respectively [33]. It is important to note that the current study’s critical finding suggests that exopolysaccharides play a role in multiple activities.

Furthermore, EPS from *Acinetobacter* sp. was characterized by using different techniques such as FTIR, a steadfast technique in identifying functional groups of biopolymer and other substances. FTIR analysis of EPS-P1 showed a variety of peaks; peaks between 1000–1200 cm^−1^ were confirmatory peaks of the polysaccharide nature of the material [33,34]. Determination of the monomer composition is the first step to understanding how exopolysaccharides act in different biological functions [41]. *Pseudomonas* sp., PT-8 produced heteropolysaccharides composed of mannose, xylose, arabinose, rhamnose, galactose, and glucose [42]. *Pseudomonas fluorescens* also produced heteropolysaccharides composed of rhamnose, fructose, galactose, and glucose [43]. It was concluded that glucose is among typical monomers in almost all types of exopolysaccharides produced by *Pseudomonas* sp. [44] reported that stress circumstances amplify the ratio and composition of monomers in the EPS compared to a non-stress environment and that glucose is a critical element in both scenarios. Therefore, it can be concluded that EPS from *Acinetobacter* sp. was quite different from exopolysaccharides reported in the literature.

EPSs are widely used as natural thickeners, stabilizers, emulsifiers, and texturizers in the industrial production of fermented milk and cheese. Additionally, they serve as food additives and functional food ingredients [45]. Recently, mutagenesis breeding and optimization of culture conditions have led to improved EPS production in laboratories [46]. Molecular evolution resulting from cold adaptation can produce unique genomic signatures in organisms, as evidenced by substitutions of specific amino acids that impact protein structure at low temperatures. However, psychrophilic strains have this ability to survive extremely low temperatures, and for this reason, they have adapted different mechanisms; for example, cold adaptation can produce unique genomic signatures in organisms, as evidenced by substitutions of specific amino acids that impact protein structure at low temperatures. For instance, arginine, which forms hydrogen bonds in protein secondary structures, can impede protein flexibility and optimal function in cold environments [47]. Hence, cold-adapted organisms may replace arginine with lysine to decrease structural stability, as seen in *Alteromonas haloplanctis*; on the other hand, some bacteria such as CUI-P1 *Acinetobacter* sp. have eight cold shock protein genes and two genes for exopolysaccharide production, so in presence of sucrose in medium, several metabolic pathways seem to be activated which resulted in higher production of EPS under optimized conditions. The genes responsible for EPS synthesis can be found on plasmids or chromosomes. In Lactococcus lactis NIZO B40, a 12 kb gene cluster on a 40 kb plasmid contains all the essential genes for EPS biosynthesis, whereas in Streptococcus thermophiles Sfi6, a 15.25 kb region encoding 16 open reading frames located on the chromosome is associated with EPS synthesis [48]. Several studies have demonstrated that overexpressing specific genes or gene clusters can enhance EPS production. For example, overexpressing the complete eps gene cluster in *L. lactis* was found to increase EPS production levels [49,50].

Moreover, it is essential to mention that *Pseudomonas fluorescens* polysaccharide reported by [43], and currently reported *Acinetobacter* sp. EPS-P1 are heteropolysaccharides and both possess glucose and galactose in their composition. Almost similar results were reported for KF5 exopolysaccharide isolated from *Lactobacillus plantarum* [51]. Similarly, *Bacillus tequilensis* FR9 exopolysaccharide exhibited a rough surface [36].

The whole genome of the *Acinetobacter* sp. possessed the most genes functioning in EPS, defense mechanisms, lipid transport, and metabolism. This genomic analysis of strain CUI-P1 provided more clues to explain the function of the strain and consequently expanded its application as a functional agent.

## 5. Conclusions

The identification of high lipase, protease, cellulase, amylase, and urease genes with glycosyltransferases (GTs) and EPS suggests potential biotechnological applications such as enzymology, ambient-temperature waste treatment, medicine, and the food industry. Specifically, the current study suggests that *Acinetobacter* sp. EPS could be utilized for cryoprotection, a crucial tool in modern biotechnology. In a nutshell, our study highlights the significant role of EPS in enhancing bacterial survivability during freeze–thaw cycles. These findings underscore the importance of further research into the underlying mechanisms and potential applications of EPS in various fields, including biotechnology, environmental science, and medicine.

## Figures and Tables

**Table 1:** Biochemical identification of strains isolated from Passu and Pisan glaciers of Pakistan.

Sample ID	Source	Biochemical Identification	EPS Production
CUI-P1	Pissan Glacier	*Acinetobacter* sp.	Yes
CUI-1A	Passu Glacier	*Exiguobacterium indicum*	Yes
CUI-1B	Passu Glacier	*Hymenobacter* sp.	Yes
CUI-1C	Passu Glacier	*Taxeobacter* sp.	No
CUI-1D	Passu Glacier	*Serratia* sp.	No
CUI-2A	Passu Glacier	*Enterobacter* sp.	No
CUI-2B	Passu Glacier	*Aeromonas hydrophila*	No
CUI-2C	Passu Glacier	*Enterococcus* spp.	No
CUI-2D	Passu Glacier	*Staphylococcus lantus*	Yes
CUI-2E	Passu Glacier	*Acinetobacter* spp.	No
CUI-2F	Passu Glacier	*Lactobacillus* spp.	No
CUI-2G	Passu Glacier	*Aeromonas hydrophila*	No
CUI-3A	Passu Glacier	*Vibrio cholerae*	No
CUI-4A	Passu Glacier	*Sphingomonas* sp.	Yes
CUI-4B	Passu Glacier	*Pseudomonas gessardii*	No
CUI-4C	Passu Glacier	*Bacillus subtilis*	No
CUI-4D	Passu Glacier	*Acinetobacter baumannii*	No
CUI-5A	Pissan Glacier	*Flavobacterium* spp.	No
CUI-5B	Pissan Glacier	*Pseudomonas* sp.	No
CUI-5C	Pissan Glacier	*Pseudomonas* sp.	No
CUI-7A	Pissan Glacier	*Aeromonas hydrophila*	No
CUI-7B	Pissan Glacier	*Serratia* sp.	No
CUI-8C	Pissan Glacier	*Pseudomonas aeruginosa*	No
CUI-9A	Pissan Glacier	*Serratia marcescens*	No
CUI-9C	Pissan Glacier	*Aeromonas* sp.	No

**Table 2:** Emulsifying activity of CUI-P1 exopolysaccharide (EPS).

Emulsifier	Incubation Time (min)	Optical Density A_540nm_	Emulsifying Activity %
**Standard**	0	0.17	100
	30	0.069	40
	60	0.032	18
	90	0.019	11
**EPS**	0	0.17	100
	30	0.166	97.6
	60	0.162	95
	90	0.141	87

**Table 3:** Genomic feature of *Acinetobacter* strain CUI-P1.

Name of Strain	*Acinetobacter* Strain CUI-P1	*Acinetobacter baumannii* ATCC 17978
**Genomic Size (Mb)**	19.5	4.09
**GC Content (%)**	42.9	38.9
**Number of Genes**	9718	3575
**Number of RNAs**	166	11
**Contigs**	199	3

## Data Availability

This Whole Genome Shotgun project has been deposited at DDBJ/ENA/GenBank under the accession JABCUD000000000. The version described in this paper is version JABCUD000000000.
